# **Real-world evidence of febrile neutropenia**-**related hospitalization on patients with perioperative chemotherapy for early breast cancer in Japan**

**DOI:** 10.1007/s12282-025-01714-6

**Published:** 2025-05-19

**Authors:** Tetsuhiro Yoshinami, Nobuhiro Shibata, Kentaro Tamaki, Kentaro Ishimaru, Satoru Ito, Tomoyuki Nukada, Shinji Ohno

**Affiliations:** 1 Department of Breast and Endocrine Surgery, Graduate School of Medicine, Osaka University, 2-2-E10 Yamadaoka, Suita City, Osaka 565-0871 Japan; 2 Department of Clinical Oncology, Kansai Medical University Hospital, 2-3-1 Shinmachi, Hirakata City, Osaka 573-1191 Japan; 3 Cancer Treatment Center, Kansai Medical University Hospital, 2-3-1 Shinmachi, Hirakata City, Osaka 573-1191 Japan; 4 Department of Breast Surgery, Nahanishi Clinic, 2-1-9 Akamine, Naha City, Okinawa 901-0154 Japan; 5 Department of Medical Affairs, Kyowa Kirin Co., Ltd., 1-9-2 Otemachi, Chiyoda-Ku, Tokyo 100-0004 Japan; 6 Department of Breast Surgery, Sagara Hospital, 3-31 Matsubara Cho, Kagoshima City, Kagoshima 892-0833 Japan

**Keywords:** Early breast cancer, Febrile neutropenia-related hospitalization, Shared decision-making, Granulocyte-colony stimulating factor, Database study

## Abstract

**Purpose:**

To clarify particularly how febrile neutropenia-related hospitalization (FNH) affects patients’ daily lives, by analyzing real-world data on FNH among patients with early breast cancer (EBC) receiving perioperative chemotherapy in Japan.

**Methods:**

This retrospective nationwide large-scale database study was conducted using anonymized claims data from 2010 to 2020. The patients with EBC who had available surgical records were included. Men, those aged < 18 years, and those who had not available chemotherapy records were excluded. FNH was defined as hospitalization during perioperative chemotherapy for EBC, with administration of intravenous antibacterial drugs and a diagnosis of FN, sepsis, infection, or fever.

**Results:**

The analysis population included 33,310 EBC patients with a mean age of 56.9 years, who received a total of 267,535 perioperative chemotherapy cycles. FNH occurred in 1,910 patients (5.73%) and 2144 chemotherapy cycles (0.80%). Median duration of FNH was 6.0 days. Fourth-generation cephalosporins were the most used intravenous antibacterial drugs (50.42%). Median duration of intravenous antibacterial drugs administration was 4.0 days. Therapeutic granulocyte-colony stimulating factor (G-CSF) was used in 1285 patients (67.28%). Median cost for FNH was estimated to be 189 thousand yen in 1,474 chemotherapy cycles with FNH, in which patients received intravenous antibacterial drugs administration for 3–8 days.

**Conclusion:**

This nationwide real-world data analysis revealed the incidence, duration, treatment patterns, and medical cost of FNH in patients with EBC receiving perioperative chemotherapy in Japan. These findings indicate that FNH imposes a considerable burden on patients’ daily lives, including time and financial impacts, contributing to the implementation of appropriate shared decision-making for primary G-CSF prophylaxis.

**Supplementary Information:**

The online version contains supplementary material available at 10.1007/s12282-025-01714-6.

## Introduction

Perioperative chemotherapy is an effective systemic therapy for preventing a relapse in patients with early breast cancer (EBC) [1–5]. Febrile neutropenia (FN) is one of the serious adverse events associated with myelosuppression by the perioperative chemotherapy [6–8]. FN also requires hospitalization in some patients, and a mortality risk of FN-related hospitalization (FNH) is reported to be 2.0 ~ 25.2% in patients with blood cancers and solid tumors, although there are variations depending on the type of cancer and treatment regimen [9–11]. FNH greatly forces restrictions on patients’ daily lives, even if an outcome of the FNH does not lead to death. Furthermore, FNH places a significant cost burden on patients [12].

The guidelines in and outside Japan recommend primary granulocyte-colony stimulating factor (G-CSF) prophylaxis for patients with a risk of FN incidence, considering the disease condition, regimen, and patients related risk factors [13–15]. Primary G-CSF prophylaxis is effective for preventing FN or FNH [16, 17], however, it is also a burden on patients in terms of medical costs, time taken for the treatment, visiting to hospital and adverse events.

Shared decision‐making (SDM) is important for high-quality cancer care, and oncologists are expected to support all patients in making informed medical decisions consistent with their needs, values, and preferences [18]. Previous qualitative analyses have shown that patient considerations for making treatment decisions are broader, incorporating the importance of efficacy and physical side effects, as well as contextual factors such as the logistics of treatments, personal and family responsibilities, and the ability to attend important events [19].

FNH occurrence is one of the important factors in SDM for using or not using a primary G-CSF prophylaxis in EBC therapy, and it is necessary for appropriate SDM to understand the real-world evidence regarding the burden of FNH on patients. There are a lot of previous findings regarding the FN, but previous reports on the FNH are limited. Especially, there are no reports specifically focused on real-world patients’ burden such as the physical or economic impact of FNH on the patients, as well as specific limitations of their daily lives caused by the FNH in patients with EBC in Japan.

Therefore, to clarify particularly how FNH affects patients’ daily lives, we analyzed real-world data on FNH among patients with EBC receiving perioperative chemotherapy in Japan.

## Methods

### Study design

This retrospective nationwide large-scale database study was conducted using anonymized and secondary data extracted from a health claims database in Japan. Data from 1 January 2010 to 31 October 2020 were analyzed. This study aimed to analyze real-world data on FNH, an event that highly affects patients’ lives, among patients with EBC receiving perioperative chemotherapy in Japan, describing the incidence and duration of FNH, use of intravenous antibacterial drugs and therapeutic G-CSF for FNH, and the medical cost for FNH.

The study protocol was approved by the Research Institute of Healthcare Data Science (RI2021015). According to the “Ethical Guidelines for Medical and Health Research Involving Human Subjects”, a study using secondary data stored in an anonymized structured format, such as this study, is outside the scope of the guidelines. Informed consent was not required for this study. This study was registered in the University Hospital Medical Information Network Clinical Trial Registry (UMIN000046199).

### Data source

This study used the Japanese Medical Data Vision (MDV) health claims database (Medical Data Vision; Tokyo, Japan), a hospital-based database, recognized as one of the largest and most credible commercially available medical databases in Japan. The MDV database contains health insurance claims data, diagnosis and procedure combination (DPC) data, and administrative data from over 400 hospitals, which covers over 30-million patients, and approximately 24% of nationwide medical institutions with emergency departments participating in the DPC/per diem payment system (as of October 2020). The data include demographics (e.g., age and sex), diagnosis codes (International Statistical Classification of Diseases and Related Health Problems, 10 th Revision [ICD-10], and disease code), Anatomical Therapeutic Chemical (ATC) code, claims, laboratory data (for some institutions) of both inpatients and outpatients, and medical fee related to hospitalization.

### Patient selection

The included patients had records of breast cancer (BC) diagnosis (ICD-10: C50) and surgery in the month of the first BC diagnosis or later identified between 1 January 2010 and 30 April 2020 in the database. The excluded patients were < 18 years old at BC diagnosis, male, with records of cancer diagnosis other than BC and chemotherapy or radiotherapy within 1 year before the first BC diagnosis, and without chemotherapy records. FNH occurs as an adverse event of chemotherapy, and therefore the patients who did not receive chemotherapy were excluded from the study. The selected patients’ records identified until 31 October 2020 were followed-up and analyzed for outcomes.

### Outcomes

The outcomes included the incidence and duration of FNH, category/name and duration of intravenous antibacterial drug used for FNH, use and duration of therapeutic G-CSF administered for FNH, and medical cost for FNH in patients receiving perioperative chemotherapy for EBC in real-word clinical practice in Japan. FNH was defined as a hospitalization during the perioperative chemotherapy for EBC, with the administration of intravenous antibacterial drugs and diagnosis of FN, sepsis, infection, or fever. For this definition, intravenous antibacterial drug was identified using ATC codes of J01 A-H, J01 K, J01P, and J01X. FN was identified using ICD-10 code of D70 and disease code of 8842350, sepsis was identified using ICD-10 codes of A02, A32, A39-41, B37, I30, I33, J02, J20, L02, L08, M86, O85, T81, infection was identified using ICD-10 codes of A01-09, A15-19, A23, A25-28, A30-32, A35-43, A46, A48, A49, B35-38, B42-49, and B99, and fever was identified using ICD-10 code of R50. The duration of FNH was defined as the period from initiation of first intravenous antibacterial drugs administration to hospital discharge. The kind and duration of intravenous antibacterial drugs used for FNH were analyzed in this study. G-CSF included filgrastim, lenograstim, nartograstim or pegfilgrastim. Prophylactic G-CSF use was defined as the single or multiple G-CSF administration, which was initiated before day 5 of each chemotherapy cycle. Therapeutic G-CSF use was defined as the G-CSF administration during the FNH, excluding the prophylactic G-CSF use. Medical cost for FNH was estimated using an amount of medical fee billed for FNH, in which the patients received intravenous antibacterial drugs administration for 3–8 days (this period was set to exclude an outlier of the estimation). Incidence of FNH with or without prophylactic G-CSF was evaluated in the first chemotherapy cycle using anthracycline or docetaxel included regimens to avoid the influence of dose reduction due to the adverse event not associated with the FNH, and restrict to the chemotherapy regimens in which prophylactic G-CSF use may be considered.

### Statistical analysis

We descriptively analyzed the data included in a claims database, and no statistical hypothesis tests were conducted in this study. The incidences of FNH were evaluated by calculating the percentage of patients with FNH among all evaluated patients, or percentage of chemotherapy cycles with FNH among all evaluated cycles. To evaluate the FNH duration, the number of FNH days was tabulated in each chemotherapy cycle, summary statistics of mean ± standard deviation (SD) and median (minimum ~ maximum) were calculated, and the number of cycles with FNH was shown in the graph stratifying into each day of hospitalization. The number of chemotherapy cycles with FNH were tabulated by each intravenous antibacterial drug class or each drug. To evaluate the duration of intravenous antibacterial drugs administration for FNH, the number of the drug administration days was tabulated in each chemotherapy cycle, summary statistics were calculated, and the number of cycles with the drug administration was shown in the graph stratifying into each administration days. Medical fee billed for FNH was tabulated in each chemotherapy cycle, the summary statistics were calculated, and the number of the cycles was shown in graph stratifying into six categories of medical cost for FNH; 0–100, > 100–200, > 200–300, > 300–400, > 400–500, and > 500 (unit: 1,000 yen). For the evaluation of therapeutic G-CSF use, the percentage of patients receiving G-CSF among all evaluated patients, or percentage of chemotherapy cycles with G-CSF use among all evaluated cycles were calculated. The incidences of FNH in the presence or absence of prophylactic G-CSF were evaluated by calculating percentage and 95% confidential interval (CI) of the number of chemotherapy cycles with FNH among all evaluated cycles.

Missing data were not imputed, and all statistical analyses were performed using SAS Release 9.4 or later (SAS Institute, NC, USA).

## Results

### Study population

Patient flow diagram is shown in supplementary figure 1. BC diagnosis records were identified in 416,455 patients in the MDV database. Of these patients, 127,287 satisfied the inclusion criteria, and 93,977 were excluded, most of whom were not treated with chemotherapy (*n *= 89,629). The analysis population comprised 33,310 patients. The mean (±SD) age of the overall population was 56.9 (±11.6) years, and most patients were <65 years old (70.43%) (Table 1). Most of the patients had no comorbidities that may be associated with infection (99.38%). As for the chemotherapy, 76.44% of the patients received anthracyclines (doxorubicin- or epirubicin-containing regimens), and 65.10% received docetaxel-containing regimens. Approximately one-third of the patients received administration of prophylactic G-CSF (36.51%).

**Table 1:** Patient characteristics

Variables	Category	Number of patients	
		*n*	(%)
Total		33,310	(100)
Age, year	Mean ± SD	56.9 ± 11.6	
	Median (min ~ max)	57.0	(20 ~ 92)
	< 65	23,460	(70.43)
	≥ 65	9,850	(29.57)
Number of comorbidity^a^ within 1 year before BC diagnosis	0	33,104	(99.38)
	1	191	(0.57)
	≥ 2	15	(0.05)
Cardiovascular disease	No	33,285	(99.92)
	Yes	25	(0.08)
Renal disease	No	33,306	(99.99)
	Yes	4	(0.01)
Liver disease	No	33,224	(99.74)
	Yes	86	(0.26)
Diabetes	No	33,204	(99.68)
	Yes	106	(0.32)
HIV or AIDS	No	33,310	(100)
	Yes	0	
Chemotherapy regimens^b^ (contains duplicates)	Anthracyclines	25,462	(76.44)
	Docetaxel	21,685	(65.10)
	Paclitaxel	11,832	(35.52)
	Others	246	(0.74)
Timing of chemotherapy	Before surgery	12,718	(38.18)
	After surgery	20,592	(61.82)
Administration of prophylactic G-CSF	No	21,150	(63.49)
	Yes	12,160	(36.51)

*AIDS* acquired immunodeficiency syndrome, *BC* breast cancer, *G-CSF* granulocyte-colony stimulating factor, *HIV* human immunodeficiency virus, *max* maximum, *min* minimum, *SD* standard deviation

^a^The comorbidity included cardiovascular disease, renal disease, liver disease, diabetes, and HIV or AIDS

^b^Anthracyclines: doxorubicin- or epirubicin-containing regimens, Docetaxel: docetaxel-containing regimens, Paclitaxel: paclitaxel- or nab-paclitaxel-containing regimens Docetaxel + doxorubicin + cyclophosphamide was counted as both groups of anthracyclines and docetaxel. Molecular targeted agents, such as trastuzumab or pertuzumab, were not counted

### Incidence and duration of FNH

**Fig. 1:**
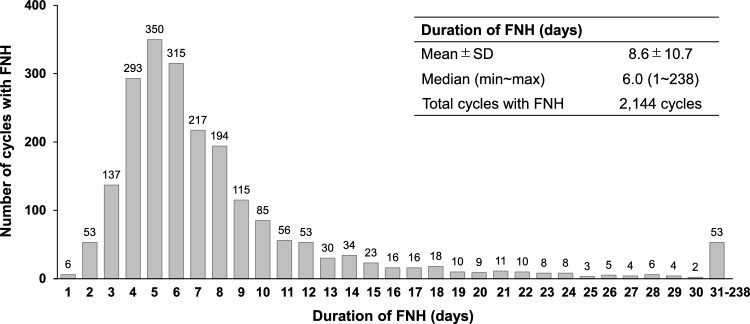
Duration of FNH. *FNH* febrile neutropenia-related hospitalization, *max* maximum, *min* minimum, *SD* standard deviation

FNH occurred in 1910 patients and 2144 chemotherapy cycles, having the incidence of 5.73% in the total evaluated patients and 0.80% in the total evaluated chemotherapy cycles, respectively (Table 2). The mean (±SD) duration of FNH in the 2144 chemotherapy cycles was 8.6 (±10.7) days, and the median (minimum ~ maximum) duration was 6.0 (1~238) days (Fig. 1). A total of one, two, and three or more times of FNH episodes was experienced in 1715, 164, and 31 patients, respectively. Overall, 17 patients (0.89%) died among 1,910 patients who had FNH, although the reason of death could not be specified in this database study.

**Table 2:** Incidence of FNH

	Patients	Cycles
	*n* = 33,310	*n* = 267,535
FNH		
Number	1910	2144
Percentage	5.73	0.80

*FNH* febrile neutropenia-related hospitalization

### Intravenous antibacterial drugs used for FNH

**Fig. 2:**
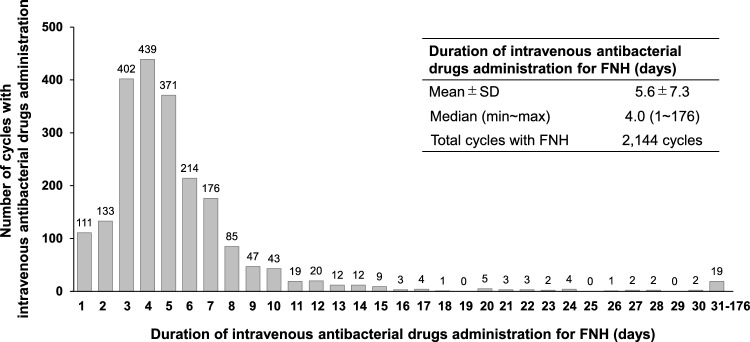
Duration of intravenous antibacterial drugs administration for FNH. *FNH* febrile neutropenia-related hospitalization, *max* maximum, *min* minimum, *SD* standard deviation

Fourth generation cephalosporins group was most commonly used as intravenous antibacterial drugs in the chemotherapy cycles with FNH (50.42%), followed by carbapenems group (22.57%) and penicillins with β-lactamase inhibitors group (19.59%) (Table 3). The specific drugs used for FNH are shown in supplementary table 1. The mean (± SD) duration of intravenous antibacterial drugs administration in the chemotherapy cycles with FNH was 5.6 (± 7.3) days, and the median (minimum~ maximum) duration was 4.0 (1~176) days (Fig. 2). Overall, among the 2,144 chemotherapy cycles with FNH, only one intravenous antibacterial drug was used in the 1,882 cycles (87.78%), 2 drugs were used in the 202 cycles (9.42%), and three or more drugs were used in the 60 cycles (2.80%).

**Table 3:** Intravenous antibacterial drugs used for FNH (by drug class)

Category name (Including multiple drug use)	Number of cycles	
	*n*	(%)
Total	2144	(100)
Fourth generation cephalosporins group	1081	(50.42)
Carbapenems group	484	(22.57)
Penicillins with β-lactamase inhibitors group	420	(19.59)
Third generation cephalosporins group	130	(6.06)
First generation cephalosporins group	117	(5.46)
Quinolone group	77	(3.59)
Second generation cephalosporins group	76	(3.54)
Glycopeptide group	40	(1.87)
Others	51	(2.38)

*FNH* febrile neutropenia-related hospitalization

### Use of therapeutic G-CSF for FNH

Among the 1910 patients who experienced FNH, therapeutic G-CSF administration was given to 1285 patients (67.28%). Among 2144 chemotherapy cycles with FNH, therapeutic G-CSF was used in 1433 cycles (66.84%). Most (72.09%) of therapeutic G-CSF was used with an administration duration of 3 days or less.

### Medical cost for FNH

**Fig. 3:**
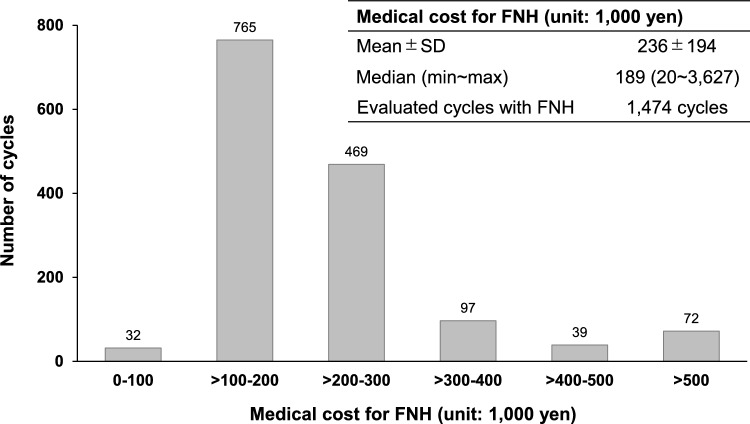
Medical cost for FNH*. FNH* febrile neutropenia-related hospitalization, *max* maximum, *min* minimum, *SD* standard deviation

In this study, the amount of medical fee billed for FNH was estimated using 1474 chemotherapy cycles with FNH, in which patients received intravenous antibacterial drugs administration for 3–8 days. An estimated 1474 cycles comprised approximately 70% of the total number of chemotherapy cycles with FNH (2144 cycles), and the median duration of FNH was 6.0 days in the 1474 cycles for this analysis, with the same duration in the 2144 chemotherapy cycles (Fig. 1). The mean (±SD) medical cost for FNH was 236 (±194) thousand yen, and the median (minimum~ maximum) medical cost was 189 (20~3627) thousand yen (Fig. 3).

### Incidence of FNH with or without prophylactic G-CSF

The incidence of FNH was evaluated during the first chemotherapy cycle using anthracycline or docetaxel included regimens with or without prophylactic G-CSF (Supplementary figure 2). A total number of the evaluated cycles was 29,946 cycles consisting of 7,029 cycles with prophylactic G-CSF and 22,917 cycles without the G-CSF. The incidence was 1.62% (114/7,029 cycles, 95% CI: 1.33~1.92%) with prophylactic G-CSF, and 3.32% (760/22,917 cycles, 3.08~3.55%) without the G-CSF.

## Discussion

In this study, the analysis population comprised 33,310 patients with 267,535 chemotherapy cycles, and a mean age of 56.9 years. The mean age of onset of BC in Japan is reported to be 60.2 years from the annual data and an analysis of the Breast Cancer Registry for 2017, which include data of the 95,203 registered Japanese BC patients from 1427 institutes [20]. The included patients were restricted to women who received perioperative chemotherapy for their EBC in this study, and therefore, the patient population is likely to be younger than the reported mean onset age of Japanese patients population with BC.

The incidence of FNH was 5.73% in all evaluated patients and 0.80% in all evaluated chemotherapy cycles, indicating a lower incidence in chemotherapy cycle-based evaluation. In this study, chemotherapy cycle using regimens with a lower risk of FN, such as weekly paclitaxel, were also included for the analysis of number of the cycles with FNH, and it is thought to result in the lower incidence in chemotherapy cycle-based evaluation. Previously, another study demonstrates that the incidences of FNH are 1.6 ~ 10.7% in EBC patients who received perioperative chemotherapy in Japan [21]. A database study conducted in the US reveals that incidence of FNH is 2.6% among BC patients who received chemotherapy in 2015 [22], although there are many limitations to the comparison of the incidence of FNH in our study in Japan and in the US because of the difference of medical system condition. This study revealed that the median duration of FNH was 6.0 days. Recently, there is growing recognition of the impact of time toxicity on patients who receive cancer treatment [23]. Our study specifically showed the time toxicity due to the FNH that may interfere with EBC patients’ lifestyle for almost a week. These findings indicate that in real-world clinical practice in Japan, FNH affects the patients’ daily lives. Consideration of FNH incidence as well as its duration is important for the appropriate management and SDM in the EBC clinical practice.

The median duration of intravenous antibacterial drugs administration in the FNH was 4.0 days, and fourth generation cephalosporins group was most commonly used for FNH therapy (50.42%), followed by carbapenems group (22.57%) and penicillins with β-lactamase inhibitors group (19.59%). The guideline on FN published by the Japanese Society of Medical Oncology recommends the empiric intravenous antimicrobial therapies for FN using antipseudomonal and broad antibacterial spectrum drugs such as cefepime (fourth generation cephalosporins group), meropenem (carbapenems group), tazobactam–piperacillin (penicillins with β-lactamase inhibitors group) [24]. The guideline also recommends the use of antibacterial drug with a minimum length required for the therapy to prevent an emergence of drug-resistant strains of bacteria [24]. Based on our findings, the guideline-recommended antimicrobial therapies are thought to be conducted for the EBC patients with FNH in the clinical practice in Japan. In addition, only one intravenous antibacterial drug was used for most of the FNH occurrence (87.78%). Unnecessary use of antibacterial drug should be avoided for preventing an emergence of drug-resistant strains of bacteria. Summarizing the findings on intravenous antibacterial drugs administration in the FNH, appropriate antimicrobial therapies are thought to be conducted in the EBC clinical practice in Japan.

To our knowledge, this is the first report on medical cost for FNH in a real-world EBC practice in Japan. The median cost for FNH was 189 thousand yen in 1474 chemotherapy cycles with FNH, in which the patients received intravenous antibacterial drugs administration for 3–8 days. We hope that FNH information obtained from this study will be made widely available to healthcare professionals so that they can use the data as one of the SDM resources for primary G-CSF prophylaxis. Therefore, it is necessary to obtain information on typical FNH that can be easily utilized for as many FNH patients as possible. However, large variations are thought to arise with regard to medical costs, because of the inclusion of extremely mild patients with doubtful FNH or, conversely, extremely severe patients with rare FNH requiring a long-term ICU use and mechanical ventilator use. To exclude these possibilities in medical cost estimation, the duration of intravenous antibacterial drugs administration was narrowed down to 3–8 days in this estimation, and this covered approximately 70% of the total case as described in the result section. In fact, the empiric intravenous antimicrobial therapies for FN using antipseudomonal and broad antibacterial spectrum drugs for 3–4 days are mentioned in the Japanese guideline [24], and about 90% of patients admitted to the ICU is reported to leave the ICU within 1 week in clinical practice in Japan [25]. Based on these supporting data, we believe that 3–8 days duration of intravenous antibacterial drugs administration is common and optimal in patients with FNH. In this common course of FNH, the patients are forced to bear a significant burden on their medical cost, and we think that these real-world data are useful for SDM about primary G-CSF prophylaxis in EBC therapy in Japan.

Among the EBC patients who experienced FNH, therapeutic G-CSF was given to 67.28% of the patients. The two Japanese guidelines do not recommend therapeutic G-CSF for cancer patients with FN [13, 24]. A questionnaire survey among hematologist and oncologist on evaluation for penetration and compliance of the Japanese guideline on FN reveals that 64.6% of respondents use G-CSF for therapeutic purposes in an FN patient “always” or “more than half” [26]. Consistent with this survey result, our study revealed that therapeutic G-CSF, which is not recommended by the clinical practice guidelines in Japan, was also used in many EBC patients with FNH, suggesting the need of education for oncologists involved in breast cancer treatment in Japan. In this study, incidence of FNH with or without prophylactic G-CSF was evaluated in the first chemotherapy cycle using anthracycline or docetaxel included regimens, and a numerically lower incidence of FNH was observed in the presence of prophylactic G-CSF compared with in the absence of the prophylactic G-CSF (1.62% [95% CI: 1.33 ~ 1.92%] vs. 3.32% [3.08 ~ 3.55%]). Prophylactic use of G-CSF has been shown to reduce FN incidence [17, 27–30]. In terms of FNH, G-CSF prophylaxis in BC patients has been reported to reduce the risk of FNH [16, 31, 32]. Therefore, prophylactic G-CSF use, according to the guidelines [13, 24], may reduce the incidence of FNH.

In this study, mortality in EBC patients who experienced FNH during perioperative chemotherapy was first reported in real-world clinical practice in Japan, and 17 deaths (0.89%) were observed among 1910 EBC patients who had FNH in the database, although the reason of death could not be specified. A US database study demonstrates that the mortality risk of FNH is 2.8% in patients with blood cancers and solid tumors [9]. The difference in the mortality risk of FNH in the two database studies may come mainly from the differences in the cancer types of the study patients, and the environment of medical care between Japan and the US.

**Fig. 4:**
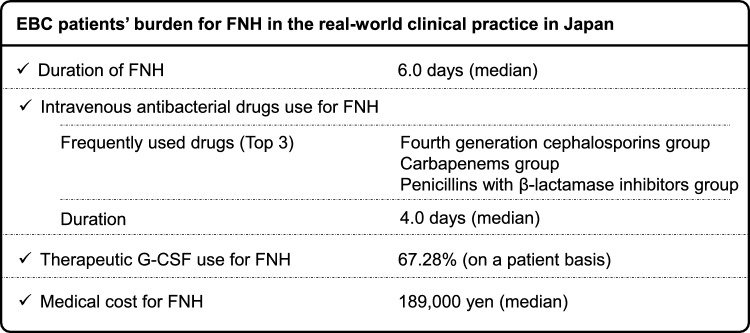
Key takeaway messages from this study*. EBC* early breast cancer, *FNH* febrile neutropenia-related hospitalization, *G-CSF* granulocyte-colony stimulating factor

We believe that the findings obtained from the current study, based on real-world data, will provide some useful information for medical providers, who are involved in clinical practice of EBC therapy. Therefore, the essence of these findings was summarized as “Key takeaway messages” in Fig. 4. The findings included in the messages are useful for understanding specifically how FNH affects patients’ daily lives if the patients have FNH. We hope that “Key takeaway messages” will be shared between medical providers and patients who need to receive perioperative chemotherapy for EBC, and appropriate SDM for primary G-CSF prophylaxis will be made also considering each patient’s background, disease status, chemotherapy regimens, and patient’s requests.

This study has several limitations in terms of generalizability, validity of diagnosis/subtypes, and statistical analysis, most of which originate from the analysis of claims database study. First, in the claims database used in this study, disease diagnosis or treatment are identified by each relevant code for insurance claims, and information for the severity of disease and the reasons why that treatment regimens are selected, which may be important confounding factors for the incidence of FNH, are insufficient. In our claims database study, we could not consider these possible confounding factors. Second, for the evaluation of FNH duration, when patients with FNH were hospitalized for additional days because of an adverse event unrelated to FNH, these additional days were wrongly added to the duration of FNH, since we cannot specify individual reasons for the additional hospitalization. Third, the analyzed database mainly contained DPC data, and data from medical institutions that did not adopt DPC were not included in this database study. Finally, this study was conducted in the Japanese specific environment of medical care, including the healthcare system and drug utilization; therefore, the study findings may not be the same in other countries that have different environment of medical care from Japan. However, in this study, a large dataset consisting of more than 30,000 patients in the claims database was analyzed, and the findings obtained from the analysis have high external validity and may contribute substantially to the better understanding of the clinical implications of FNH.

In conclusion, this nationwide large-scale real-world data analysis first revealed the incidence and duration of FNH, use of intravenous antibacterial drugs and therapeutic G-CSF for FNH, and medical cost for FNH in patients with EBC in clinical practice in Japan, using a claims database. The real-world evidence suggests that patients with FNH have negative influences on their lives, including time and financial impacts. We believe that these findings will be useful for understanding particularly how FNH occurrence affects the patients’ daily lives, which contributes to the implementation of appropriate SDM for primary G-CSF prophylaxis in EBC therapy in Japan.

## Supplementary Information

Below is the link to the electronic supplementary material.

Supplementary file1 (DOCX 30 KB)

## Data Availability

The data that support the findings of this study are proprietary to Medical Data Vision Co., Ltd. (MDV), and restrictions apply to the availability of these data, which were used under license for the current study, and thus are not publicly available. The data are available for purchase from the MDV. For inquiries regarding data purchases, please contact ebm_sales@mdv.co.jp.
