# Supplementary material for: Real-world evidence of febrile neutropenia-related hospitalization on patients with perioperative chemotherapy for early breast cancer in Japan
Source: Breast Cancer. 2025 May 19;32(4):857–66. doi: 10.1007/s12282-025-01714-6 (PMC12174221; doi:10.1007/s12282-025-01714-6)
Supplement: Supplementary file 1 — Supplementary file1 (DOCX 30 KB) [file 12282_2025_1714_MOESM1_ESM.docx]

**[Supplementary figures and table]**

**Supplementary figure 1 Patient flow diagram**

*MDV* Medical Data Vision, *BC* breast cancer

**Supplementary table 1 Intravenous antibacterial drugs used for FNH (by each individual drug)**

| **Category  (including multiple drug use)** | **Name** | **Number of cycles** |
| --- | --- | --- |
|  |  | **n (%)** |
| Total |  | 2,144 (100) |
| Fourth generation cephalosporins group | Cefepime hydrochloride hydrate | 988 (46.08) |
|  | Cefozopran hydrochloride | 93 (4.34) |
| Carbapenems group | Meropenem hydrate | 458 (21.36) |
|  | Doripenem hydrate | 11 (0.51) |
|  | Imipenem hydrate･Cilastatin sodium | 10 (0.47) |
|  | Biapenem | 4 (0.19) |
|  | Panipenem･Betamipron | 1 (0.05) |
| Penicillins with β-lactamase inhibitors group | Tazobactam･Piperacillin sodium | 375 (17.49) |
|  | Ampicillin sodium･Sulbactam sodium | 45 (2.10) |
| Third generation cephalosporins group | Ceftriaxone sodium hydrate | 91 (4.24) |
|  | Ceftazidime hydrate | 23 (1.07) |
|  | Cefoperazone sodium･Sulbactam sodium | 16 (0.75) |
| First generation cephalosporins group | Cefazolin sodium | 117 (5.46) |
| Quinolone group | Ciprofloxacin | 37 (1.73) |
|  | Levofloxacin hydrate | 36 (1.68) |
|  | Pazufloxacin mesilate | 4 (0.19) |
| Second generation cephalosporins group | Cefmetazole sodium | 32 (1.49) |
|  | Flomoxef sodium | 25 (1.17) |
|  | Cefotiam hydrochloride | 19 (0.89) |
| Glycopeptide group | Vancomycin hydrochloride | 37 (1.73) |
|  | Teicoplanin | 3 (0.14) |
| Broad-spectrum penicillin group | Piperacillin sodium | 7 (0.33) |
|  | Ampicillin sodium | 5 (0.23) |
| Aminoglycoside group | Amikacin sulfate | 8 (0.37) |
|  | Gentamicin sulfate | 2 (0.09) |
| Macrolide group | Azithromycin hydrate | 8 (0.37) |
| Fosfomycin group | Fosfomycin sodium | 5 (0.23) |
| Lipopeptide group | Daptomycin | 5 (0.23) |
| Lincosamide group | Clindamycin phosphate | 5 (0.23) |
| Oxazolidinone group | Linezolid | 2 (0.09) |
| Tetracycline group | Minocycline hydrochloride | 2 (0.09) |
| ST combination group | Sulfamethoxazole･Trimethoprim | 1 (0.05) |
| Streptomycin group | Streptomycin Sulfate | 1 (0.05) |

*FNH* febrile neutropenia related hospitalization

**Supplementary figure 2 Incidence of FNH with or without prophylactic G-CSF**

Error bar expresses 95% confidential interval

*FNH* febrile neutropenia-related hospitalization, *G-CSF* granulocyte-colony stimulating factor

FNH incidence was evaluated in the first chemotherapy cycle using anthracycline or docetaxel included regimens.
